# Effect of Composition Interactions on the Dose Response of an *N*-Isopropylacrylamide Gel Dosimeter

**DOI:** 10.1371/journal.pone.0044905

**Published:** 2012-10-12

**Authors:** Yuan-Jen Chang, Bor-Tsung Hsieh

**Affiliations:** 1 Department of Management Information Systems, Central Taiwan University of Science and Technology, Taichung, Taiwan, Republic of China; 2 Institute of Biomedical Engineering and Material Science, Central Taiwan University of Science and Technology, Taichung, Taiwan, Republic of China; 3 Department of Medical Imaging and Radiological Science, Central Taiwan University of Science and Technology, Taichung, Taiwan, Republic of China; Duke University Medical Center, United States of America

## Abstract

In this study, a two-level full factorial design was used to identify the effects of the interactions between compositions in an *N*-isopropylacrylamide (NIPAM) gel dosimeter involving the following variables: (A) gelatin, (B) NIPAM, (C) the crosslinker N, N′-methylene-bis-acrylamide (Bis), and (D) the antioxidant tetrakis (hydroxymethyl) phosphonium chloride (THPC). The dose range was from 0 Gy to 5 Gy. Optical computed tomography was used to scan the polymer gel dosimeter. Each component was set to two levels for all four variables, including (A) 4% and 6%, (B) 4% and 6%, (C) 2% and 4%, as well as (D) 5 and 15 mM. Response surface methodology and a central composite design were adopted for the quantitative investigation of the respective interaction effects on the dose response curve of the gel. The results showed that the contributions of the interaction effects, i.e., AB (6.22%), AC (8.38%), AD (7.74%), BC (9.44%), ABC (18.24%), BCD (12.66%), and ABCD (13.4%), were greater than those of the four main effects, accounting for over 76.08% of the total variability. These results also indicated that the NIPAM gel recipe with the highest sensitivity was at 40%C (mass fraction of Bis).

## Introduction

The rise in the number of radiotherapies used in dosimetry cases with high-dose gradient distribution signifies the increasing challenges in dose verification [1]. A significant development in gel dosimetry was seen when Fong proposed a new type of gel dosimeter [2]. Without the drawback of ferric ion diffusion found in Fricke-type gel dosimeters, the polymer-gel dosimeter is considered the most promising among the newly developed dosimeters because of its superior spatial and temporal stability. Moreover, for high-energy photo irradiation, most polymer-gel dosimeters can be considered soft tissue equivalent [1], [3], [4]. Indeed, polymer-gel dosimetry is a promising dosimeter for dose determination in radiation therapy [5]. However, the spatial resolution and accuracy of absorbed dose measurements remain important issues in the identification of the best gel dosimetry system, i.e., the best gel and readout method to be used in medical applications. The main medical applications include high dose rate gradient radiation fields such as intensity modulated radiotherapy and stereotactic radiosurgery.

Several studies [6]–[14] have described the chemical reaction of radiation-induced polymerization in polymer-gel dosimeters. ^1^H and ^13^C-NMR spectroscopy and FT-Raman spectroscopy have been used to investigate the properties of polymer-gel dosimeter post-irradiation [6]. Upon irradiation, the polymerization process is initiated by radical products of water. This process involves water radiolysis, initiation reactions, propagation reactions, and termination reactions. The gel components show various degrees of interaction depending on their concentration. Jirasek and Duzenli [7], as well as Koeva et al. [8] have investigated the effect of the crosslinker fraction in polymer gel. Hayashi [9] has studied the role of gelatin in methacrylic-acid-based gel dosimeters. Given that oxygen (O_2_) tends to react with the free radicals generated by water radiolysis, which in turn terminates the polymerization process, a number of studies have proposed that some antioxidant additions to the gel are needed to inhibit O_2_ reaction [3], [10]–[12]. Some researchers have revealed that the dose response curve show different sensitivities using different monomers [12]–[14]. However, a variety of chemical and physical phenomena influence the radiation-induced polymerization process, including crosslinking, oxygen participation, and interaction between compositions. Therefore, a comprehensive understanding of the complicated polymerization process and gel composition interactions using a systematic approach is necessary.

Various readout methods have been suggested, such as magnetic resonance imaging (MRI), optical-CT scanning, X-ray CT, and ultrasound. Most of the previous studies focused on MRI and laser-based optical-CT scanning. For gel dosimetry, optical-CT scanning has the advantage of simplicity and cheaper implementation. However, the accurate and economical 3-D measurement of optical density (OD) is a crucial step in 3-D polymer-gel dosimetry in conjunction with optical-CT scanning. Some effective OD calibration methodologies have been investigated for fast and high-resolution CCD-based optical-CT scanning [15]–[17]. To reduce the uncertainty of optical density measurement, related factors that affect measurement in cone-beam CT have been studied [18]–[19].

Polymer-gel dosimeters become more opaque when irradiated. The characteristic curve of gel opaqueness to irradiation is the dose response, which is represented by two-gel parameters, i.e., linearity and sensitivity [20]. One important step in 3-D dose distribution reconstructed from an optical laser scanner is sensitivity calibration [21]. To accomplish this calibration, glass vials with different doses are used to obtain the linear equations of the unit length optical density value (OD/cm) corresponding to the doses delivered to each glass vial [22]–[24]. The linear equation can be used to calculate dose distribution during 3-D reconstruction. Based on the linear optical response of a gel with the dose, Wuu and Xu [25] have adopted the two-point calibration approach to obtain the relative dose distribution inside an irradiated gel. Combined with ion chamber measurements at two certain points along the central axis of treatment planning, the entire 3-D dose distribution can be obtained. Both parameters are obtained from a linear regression model of the dose response. The optical-computed tomography (optical-CT) coefficients are related to the true attenuation coefficients; thus, adopting the two-point calibration method or linear equation of the unit length optical density value may result in large errors in 3-D dose distribution if the linear relationship is inaccurate [26]. To achieve a more accurate dose distribution using optical-CT, four potential uncertainties should be considered [23]. The first and second uncertainties are the electronic and mechanical noises derived from the optical and mechanical signal acquisition processes, respectively. The third uncertainty is the image reconstruction algorithm. The final and most important uncertainty is the non-uniformities of the gel. To improve gel uniformities, chemical reactions and composition interactions in radiation-induced polymerization need to be scrutinized.

The dose response curve depend not only on initial gel compositions but also on different dose ranges [7], [27]. The dose sensitivity is lower in low doses than in high doses [1], [20]. To understand further the effects of chemical interactions between gel compositions, tetrahydroxyphenylchlorin (THPC), and gelatin, the current study examined the effects in the low-dose range, which is most commonly used in clinical applications. The quantitative contribution of each composition and its effects on the gel characteristics were investigated. The effects of the complicated chemical interaction of the composition on sensitivity and linearity during polymerization were also discussed.

## Materials and Methods

### 2.1. *N*-isopropyl acrylamide (NIPAM) polymer gel preparation and irradiation

**Figure 1:**
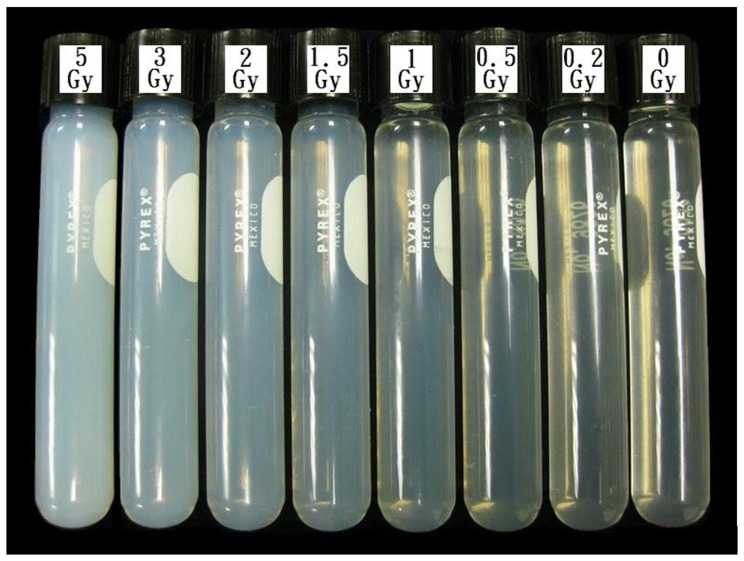
Glass vials of NIPAM gels irradiated to graded doses.

The NIPAM polymer gel used in this study was manufactured based on the procedures presented by De Deene et al. [12] and Senden et al. [14]. The gel was manufactured on a bench-top under normal atmospheric conditions using tetrakis (hydroxymethyl) phosphonium chloride (THPC) as a free radical scavenger. The prepared gel solutions were poured into Pyrex screw test vials (Pyrex Model No. 9826; 13 mm in outer diameter, 100 mm long), as shown in Fig. 1. The vials were wrapped in aluminum foil to prevent photo-polymerization by environmental light. The polymer gels were then carefully stored in a refrigerator at a fixed temperature (7°C) until complete solidification. Each gel sample was irradiated at various doses (i.e., 0, 0.2, 0.5, 1, 1.5, 2, 3, and 5 Gy) using a 6 MV linear accelerator (Varian 21× Clinac, Varian Ltd., Palo Alto, CA, USA).

Irradiation was performed less than 6 h after gel fabrication to avoid oxygen diffusion. A 13 mm-diameter hole was punctured to accommodate the placement of Pyrex test vials in the center of the short side of a customized 30×30×4 cm^3^ acrylic phantom. The non-irradiated gel vial was placed in the hole in the acrylic phantom to provide adequate build-up and scattering conditions. To ensure accurate location, two 3.5 and 16.5 cm-long acrylic sticks were placed adjacent to the upper and lower sides of the test tube, respectively. The acrylic phantom was placed between two 3 cm-long solid water phantoms to ensure that the source surface distance was 5 cm. All samples were maintained at room temperature (22°C) before irradiation.

### 2.2. Readout process by optical laser scanning

Magnetic resonance imaging (MRI) gel dosimetry allows the imaging only of arbitrarily shaped gel dosimeters in phantoms. In comparison, optical-CT can provide a low-cost solution for many applications [28], [29]. Gore et al. [28] has proposed an optical laser scanning system that incorporates a He-Ne laser, photodiode detectors, and a rotating gel platform. Their experimental results indicate better than 5% accuracy with a spatial resolution of approximately 2 mm using the current prototype scanner. Micro optical-CT has been used to study the characteristics of a high-dose gradient dosimeter [30]. To improve scanning speed, a rotating mirror and Fresnel lenses were used [31]–[33]. A significant development in optical-CT scanning was proposed by Krstajic and Doran (2007) [15]–[17]. In their study, a pair of galvanometer-controlled mirrors was used to manipulate the laser beam and the scanning speed. The commercial optical-CT scanner called Vista, which was manufactured and distributed by Modus Medical Devices, Inc. (London, Ontario, Canada), was developed to provide fast scanning speed [18]–[19].

**Figure 2:**
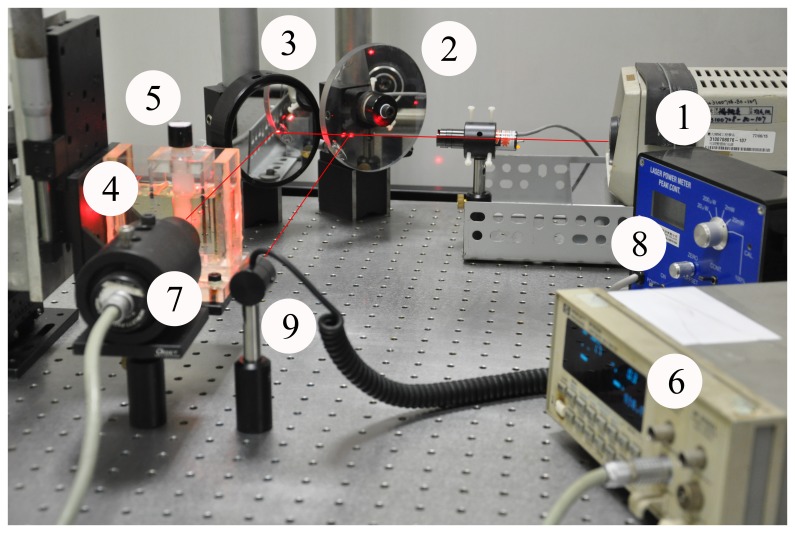
Schematic diagram of the setup for measuring the attenuation coefficient by optical laser scanning system. (1) 632.8 nm He-Ne laser, (2) beam splitter, (3) mirror, (4) oil tank, (5) gel in vial, (6) optical power meter, (7) optical sensor head, (8) optical power meter, and (9) optical sensor head.

Based on the experiment conducted by Gore et al. [28], the current study used an optical laser scanning system with a 632.8 nm He-Ne laser (20 mW). An optical power meter was used to obtain the optical density *I* of the irradiated gel, as shown in Fig. 2. A background optical intensity *I*_0_ was also obtained from non-irradiated gel vials using the same experimental parameters. Each testing vial was mounted on a precision three-axis stage and immersed in a 90×37×95 mm^3^ tank (Pyrex glass; 1 mm thick) filled with vegetable oil. To minimize refraction and reflection at the interface, the oil had a refractive index similar to that of Pyrex glass. Room temperature was maintained at 22°C using an air conditioner to avoid temperature-induced dose deviation [34]. The attenuation coefficient α of the irradiated gel was determined by the following equation [14]:
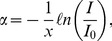
(1)where *x* is the gel diameter. The optical dose response curve, measured in terms of the attenuation coefficient and absorbed dose for each polymer gel recipe, was then obtained. The linear correlation coefficient (*R* ^2^) was evaluated from the dose response curve. The slope of the dose response curve denoted the sensitivity [8], [14], [24], [35].

### 2.3. Central composite design (CCD)

The amounts of gel components and their interactions affect the dose response of the gel [11], [14]. Chang et al. [27] have applied a statistical method using a two-level fractional factorial plan to determine the optimal composition of NIPAM gel. In the current study, a two-level factorial design of experiment in conjunction with response surface methodology and a CCD were adopted to differentiate the effects between each composition and their interactions. The basic CCD for *k* variables typically consists of a 2*^k^* factorial design with each factor at two levels, −1 and +1, superimposed on a star design, or 2*^k^* axial points and several repetitions at the design center points. The axial points are factors set to mid-values of the high and low level of the factors. Therefore, the 2*^k^* design of the experiment augmented with the center point is an excellent method that can be used to indicate curvature [36].

In this work, a 2*^k^* factorial design was used to investigate the dose response characteristics of a NIPAM gel dosimeter as a function of the following four factors: gelatin (A), NIPAM (B), Bis (C), and THPC (D). Each factor ran at two levels: gelatin, 4% and 6%; NIPAM, 4% and 6%; bis, 2% and 4%; and THPC, 5 and 15 mM (Table 1). The experimental design was a 2^4^ factorial design. The variables A, B, C, and D were defined on a coded scale from −1 to +1 (the low and high levels of A–D). To consider the curvature effect in the response function, the test for curvature was performed by adding five center points to the 2^4^ factorial designs. Finally, the regression model was represented by the terms of the coded factors [33]. Two batches of gels of the same composition were produced to replicate the experiments. The CCD design matrix was then generated and analyzed to determine the dose range of optimal compositions using the DESIGN-EXPERT software.

**Table 1:** Selected variables and experimental design levels used in the experiment.

Variables					
		Gelatin (A)	NIPAM (B)	Bis (C)	THPC (D)
Coded levels	+1	6%	6%	4%	15 mM
	−1	4%	4%	2%	5 mM

Based on the CCD design with the four independent variables (gelatin, NIPAM, Bis, and THPC) and two levels of each variable, a 2^4^ design augmented with two replicated batches and five center points yielded a total of 37 runs. The test results of the experiments are listed in Table 2. The sensitivity and linearity for the dose range of 0–5 Gy were computed. The gel composition had different effects on the sensitivity and linearity across various dose ranges.

**Table 2:** Linearity and sensitivity of the tested gels.

Sample	Experimental design variables				Linearity	Sensitivity
Gels	A (%)	B (%)	C (%)	D (mM)	0–5 Gy	0–5 Gy (Gy^−1^⋅mm^−1^)
1	4	4	2	5	0.941	0.0100
2	4	4	2	5	0.948	0.0091
3	6	4	2	5	0.947	0.0027
4	6	4	2	5	0.950	0.0024
5	4	6	2	5	0.956	0.0052
6	4	6	2	5	0.956	0.0048
7	6	6	2	5	0.985	0.0021
8	6	6	2	5	0.979	0.0020
9	4	4	4	5	0.987	0.0048
10	4	4	4	5	0.990	0.0045
11	6	4	4	5	0.943	0.0024
12	6	4	4	5	0.947	0.0022
13	4	6	4	5	0.965	0.0129
14	4	6	4	5	0.951	0.1340
15	6	6	4	5	0.967	0.0049
16	6	6	4	5	0.988	0.0047
17	4	4	2	15	0.992	0.0092
18	4	4	2	15	0.971	0.0101
19	6	4	2	15	0.985	0.0025
20	6	4	2	15	0.985	0.0024
21	4	6	2	15	0.986	0.0147
22	4	6	2	15	0.986	0.0151
23	6	6	2	15	0.975	0.0031
24	6	6	2	15	0.973	0.0029
25	4	4	4	15	0.995	0.0108
26	4	4	4	15	0.992	0.0108
27	6	4	4	15	0.995	0.0046
28	6	4	4	15	0.995	0.0043
29	4	6	4	15	0.997	0.0218
30	4	6	4	15	0.999	0.0221
31	6	6	4	15	0.989	0.0267
32	6	6	4	15	0.989	0.0248
33	5	5	3	10	0.995	0.0115
34	5	5	3	10	0.993	0.0113
35	5	5	3	10	0.993	0.0110
36	5	5	3	10	0.994	0.0113
37	5	5	3	10	0.994	0.0113

## Results and Discussion

### 3.1 Dose-response curve

**Figure 3:**
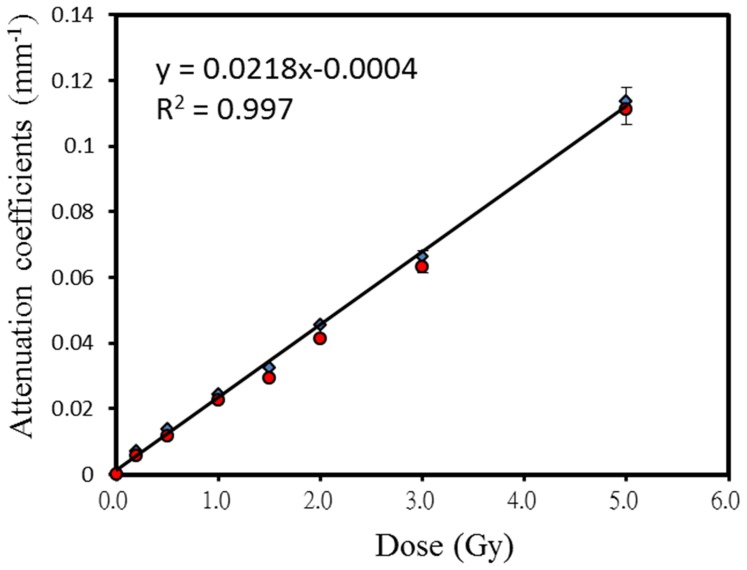
Dose-response curve of NIPAM gel obtained from the glass vial approach with 6 MV irradiation (gelatin, 4%; NIPAM, 6%; Bis, 4%; and THPC, 15 mM). The error bars indicate and experimental uncertainty of 5%.

Given that the gels gradually became more opaque upon increased irradiation dose, the light intensity decreased and led to increased attenuation coefficients (Eq. 1). The relationship of the attenuation coefficients with the dose is called the dose-response curve. Figure 3 shows the dose-response curve of NIPAM gel with 4% gelatin, 6% NIPAM, 4% bis, and 15 mM THPC. Two batches of experimental data are shown in the same graph. The graph shows that the experimental data can reproduce very well. The least square fit of the dose-response curve can be obtained as *y* = 0.0218*x*−0.0004, and the *R* ^2^ value is 0.997. Previous studies have shown that the sensitivity is the slope of the dose-response curve, i.e., 0.0218 mm^−1^Gy^−1^. On the other hand, the linearity is the goodness of fit of this experimental data for this gel composition, i.e. 0.997 [8], [9], [19], [27]. The linearity indicates the linear relationship to ensure accurate measurement. The sensitivity and linearity of all gel compositions were calculated and are listed in Table 2.

### 3.2 Statistical analysis of sensitivity and linearity

Linearity and sensitivity data were entered into the CCD design matrix, as shown in Table 2. Data were formulated using the Design-Expert software (Stat-Ease, Inc., Minneapolis, MN, USA) for the determination of significant variables. The characteristics of the dose response (linearity and sensitivity) for the dose range of 0–5 Gy are listed. In this study, a regression analysis was conducted to develop a best-fit model between the dose response (linearity and sensitivity) and the four significant variables for the dose range of 0–5 Gy. The regression analysis results showed that the regression models for linearity were significant for the dose range of 0–5 Gy (*p*<0.05) (Table 3). Thus, 2^4^ – 1 effect estimates, including 4 main effects, 6 two-factor interactions, 4 three-factor interactions, and 1 four-factor interaction, were obtained for the dose range of 0–5 Gy (Table 4). The results also showed that the contributions of the interaction effects, such as AB (6.22%), AC (8.38%), AD (7.74%), BC (9.44%), ABC (18.24%), BCD (12.66%), and ABCD (13.4%), were greater than those of the four main effects, accounting for over 76.08% of the total variability. This finding indicated that the interaction effect between factors play a more important role in the gel dose response for the dose range of 0–5 Gy.

**Table 3:** ANOVA for the dose range of 0–5 Gy.

Source	Sum of squares	Degree of freedom	Mean square	*F*	*p*
Model	1.42×10^−2^	15	9.50×10^−4^	16.67	<0.0001
A	7.47×10^−4^	1	7.47×10^−4^	13.11	0.0017
B	1.62×10^−6^	1	1.62×10^−6^	0.03	0.8678
C	3.92×10^−4^	1	3.92×10^−4^	6.88	0.0163
D	1.34×10^−3^	1	1.34×10^−3^	23.59	<0.0001
AB	1.02×10^−4^	1	1.02×10^−4^	1.79	0.1954
AC	1.10×10^−3^	1	1.10×10^−3^	19.38	0.0003
AD	1.49×10^−3^	1	1.49×10^−3^	26.11	<0.0001
BC	1.38×10^−3^	1	1.38×10^−3^	24.14	<0.0001
BD	1.68×10^−3^	1	1.68×10^−3^	29.42	<0.0001
CD	1.88×10^−4^	1	1.88×10^−4^	3.3	0.0842
ABC	1.29×10^−4^	1	1.29×10^−4^	2.26	0.1483
ABD	3.24×10^−3^	1	3.24×10^−3^	56.87	<0.0001
ACD	4.21×10^−6^	1	4.21×10^−6^	0.07	0.7887
BCD	2.03×10^−4^	1	2.03×10^−4^	3.56	0.0737
ABCD	2.25×10^−4^	1	2.25×10^−3^	39.45	<0.0001
Curvature	2.38×10^−3^	1	2.38×10^−3^	41.77	<0.0001
Pure error	1.14×10^−3^	20	5.70×10^−5^		
Corrected total	1.78×10^−2^	36			
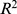 = 0.9259					
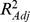 = 0.8704					

**Table 4:** Effect estimates for the dose ranges 0–5 Gy.

	0–5 Gy
Term	Contribution (%)
Model	4.20
A	0.01
B	2.21
C	7.57
D	0.57
AB	6.22
AC	8.38
AD	7.74
BC	9.44
BD	1.06
CD	0.72
ABC	18.24
ABD	0.02
ACD	1.14
BCD	12.66
ABCD	13.40
Pure Error	6.42

The regression model equations describing the relationship between sensitivity and linearity, as well as the four factors for the dose range of 0–5 Gy were as follows:
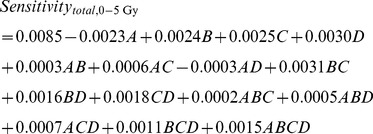
(2)

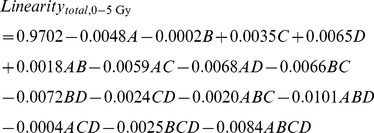
(3)For the dose range of 0–5 Gy, 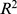 was 0.9697 and 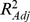 was 0.9470 for sensitivity, whereas 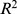 was 0.9259 and 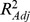 was 0.8704 for linearity (Eqs. 2 and 3), indicating that these models very well explained the experimental data.

### 3.3 Confirmation experiments

From the regression models (Eqs. 2 and 3) proposed in the previous section, several recipes with linearities greater than 0.995 were suitable to meet the clinical usage requirements. However, two better recipes and one worse recipe were specifically selected for the confirmation experiments to verify the validity of the regression models and exclusion of relatively important factors. The experimental and predicted values for these recipes were compared, and Table 5 shows the results for the dose range of 0–5 Gy. The errors between the experimental and predicted values were found to be all less than 1%. Therefore, the predictions of the regression model were valid and adequate.

**Table 5:** Comparison of predicted and experimental values for the dose range of 0–5 Gy.

		Sample			Linearity	
Gelatin	NIPAM	Bis	THPC	Predict value	Experiment value	Error (%)
4.00	5.00	4.00	15.00	0.996	0.956	0.24
					0.967	0.14
4.00	6.00	4.00	15.00	0.998	0.999	0.03
					0.998	0.13
6.00	4.00	2.00	5.00	0.949	0.956	0.18
					0.954	0.34

### 3.4 Effect of the interaction of gel compositions on the gel dose response

**Figure 4:**
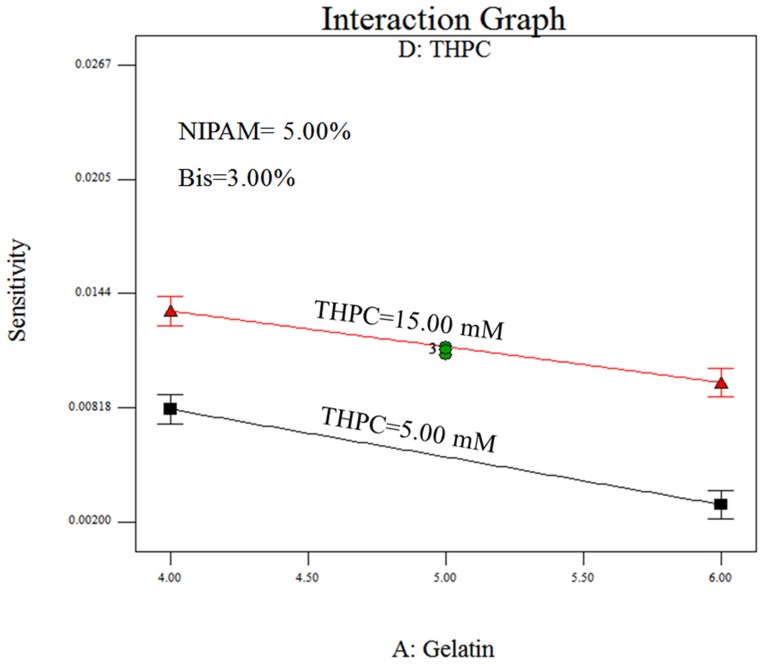
Interaction of gelatin and THPC for *sensitivity_total, 0–5 Gy_*.

For a normoxic polymer gel, antioxidant additions to the gel are needed to inhibit O_2_ reaction [2], [12], [13]. However, Jirasek et al. [7] have found that the amount of antioxidants must exceed a certain level of concentration to reach the O_2_ inhibition region. Equations. (2) and (3) indicate that the effect of THPC is positive for both sensitivity and linearity because of the positive algebraic sign for factor D. Hence, increasing the amount of THPC improves the sensitivity and linearity for the dose range of 0–5 Gy, similar to the conclusion of De Deene *et al.* (2002) [12]–[13]. A similar trend can be obtained for Bis because of the positive algebraic sign for factor C. Bis acts as a crosslinker to increase polymerization rates. Jirasek and Duzenli [9] have proposed that 30%C (mass fraction of Bis) PAGs are the most sensitive dosimeters for the low-dose range of 0–5 Gy. In the current study, gel samples 29–32 had a higher sensitivity of 40%C. Gel recipes with greater and less than 40%C showed decreased sensitivity at the same THPC concentration. A negative trend for both sensitivity and linearity was observed with increased gelatin (factor A). A similar conclusion about the effect of gelatin on sensitivity can be found in De Deene's studies [12]–[13]. This result can be explained by the interaction of gelatin with THPC, as shown in Figure 4. Increased gelatin level decreased the gel sensitivity because high-content gelatin resulted in a low polymerization rate. The gelatin also reacted with free radicals, thereby reducing the polymer reaction and decreasing the gel sensitivity, as explained by De Deene *et al.* [12], [13].

**Figure 5:**
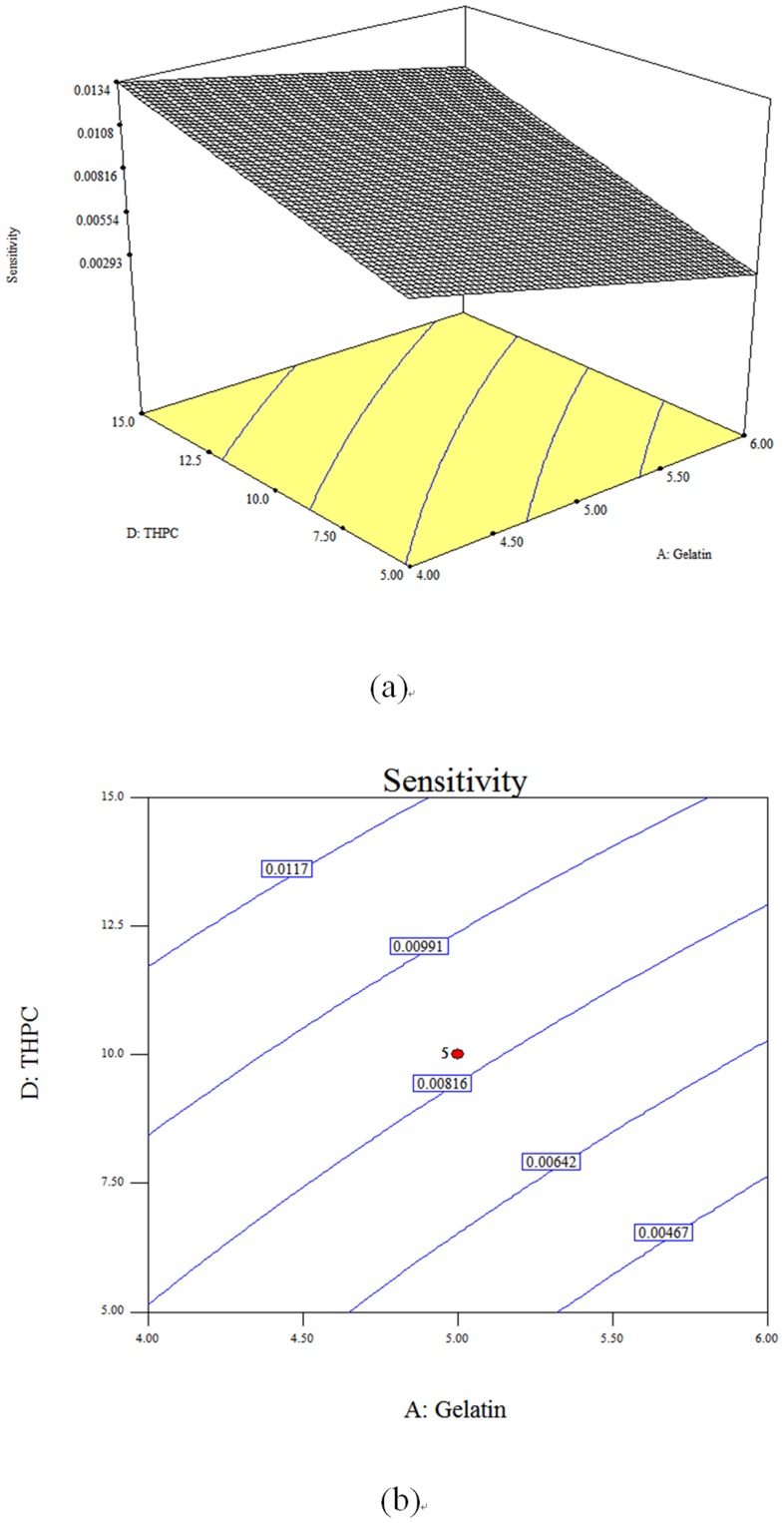
Three-dimensional response surface plot and contour plot of gelatin and THPC for *sensitivity_total, 0–5 Gy_*: (a) response surface; (b) contour plot.

Figure 5 illustrates the response surfaces and contour plots of gelatin and THPC with sensitivity for the dose range of 0–5 Gy. Regarding the interaction effects, both sensitivities decreased with increased gelatin content at THPC concentrations of 5 and 15 mM (low and high levels, respectively). However, the sensitivity decreased to 29.1% (sensitivity from 0.0134 to 0.0095 with gelatin content from 4% to 6%) when THPC = 15 mM, which was lower than 64.2% (sensitivity from 0.0081 to 0.0029 with gelatin content from 4% to 6%) when THPC = 5 mM. Thus, THPC reacted with the gelatin and reduced the amount of free radicals consumed by the gelatin [7], [13].

### 3.5 Effects of linearity and sensitivity

Clinic applications require the use of gel dosimeters with appropriate sensitivity and linearity. For some readout methods (e.g., X-ray CT), a gel recipe with higher sensitivity may be chosen. For some other higher dose range applications [25], a gel recipe with lower sensitivity may be chosen so that samples do not become too opaque. Eq. (2) is an equation for predicting the gel sensitivity within the dose range of 0–5 Gy. While determining the sensitivity of a gel, Xu et al. [22] have proposed a method to obtain optimal image contrast in the optical-CT scanning of gel dosimeters. The optical density increment is limited by the effective dynamic range of the optical detector. When the optical density increment exceeds the limitation, an artifact arises in the reconstructed image and causes a larger uncertainty in the dose estimate. The distortions of the image caused by the artifact indicate that the signals in these regions are larger than their actual values; thus, the dose in this area is over estimated. To achieve more accurate dose distribution, the gel recipe can be adjusted in advance to obtain optimal scanning results.

## Conclusion

In this study, an experimental design was adopted to investigate the characteristics of the dose-response curve in terms of sensitivity and linearity for the dose range of 0–5 Gy. The sensitivity and linearity were strongly affected by the interactions of gel composition. This study provided quantitative analyses of the interaction effects of gel compositions based on statistical analyses, and revealed that the effects on the dose-response curve were different. Based on the results, the suitable sensitivity and linearity of the gel should be adjusted in advance for different clinical applications to achieve more accurate dose distribution in 3-D image reconstruction. The error between the predicted and experimental values was also found to be less than 1% for the dose range of 0–5 Gy. The NIPAM gel recipe with the highest sensitivity was 40%C.
